# A Historical Perspective on Uremia and Uremic Toxins

**DOI:** 10.3390/toxins16050227

**Published:** 2024-05-15

**Authors:** Björn Meijers, Ward Zadora, Jerome Lowenstein

**Affiliations:** 1 Nephrology and Transplantation Unit, University Hospitals Leuven, 30000 Leuven, Belgium; bjorn.meijers@uzleuven.be (B.M.); ward.zadora@kuleuven.be (W.Z.); 2 Laboratory of Nephrology, Katholieke Universiteit Leuven, 30000 Leuven, Belgium; 3 Nephrology Division, NYU Langone Medical Center, New York, NY 10016, USA

**Keywords:** uremia, remote sensing and signaling, dialysis

## Abstract

Uremia, also known as uremic syndrome, refers to the clinical symptoms in the final stage of renal failure. The definition of the term has changed over time due to an improved comprehension of the kidney’s function and the advancement of dialysis technology. Here, we aim to present an overview of the various concepts that have developed regarding uremia throughout the years. We provide a comprehensive review of the historical progression starting from the early days of Kolff and his predecessors, continuing with the initial research conducted by Niwa et al., and culminating in the remote sensing hypothesis of Nigam. Additionally, we explore the subsequent investigation into the function of these toxins as signaling molecules in various somatic cells.

## 1. Introduction

**Figure 1:**
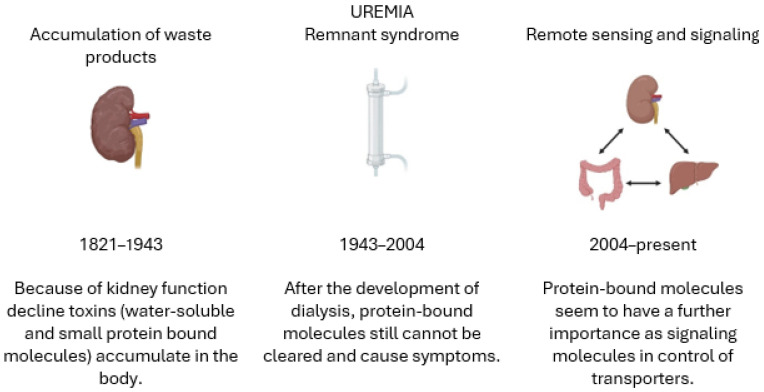
Our understanding of uremia has evolved over time. Three main periods can be recognized. The first era is considered as the nascence of uremic intoxication. The second era starts after the development of dialysis as a clinical treatment for individuals affected by kidney failure. Uremia was seen as a remnant syndrome due to the incomplete removal of accumulated toxic waste substances. The current era of uremia research is based on molecular signaling cascades.

The syndrome of uremia is ill-defined and ill-understood. One definition, provided by Meyer and Hostetter, describes uremia as an illness accompanying kidney failure that is unexplained by derangements in extracellular volume, inorganic ion concentrations, or the lack of known renal synthetic products [1]. In their excellent review, they aptly note that the meaning of uremia, or at least the prevailing paradigm, has changed over time [1]. Indeed, our understanding of what we understand as uremia and its underlying pathophysiology has shifted substantially. Historically, three different paradigms may be discerned. First, the recognition that uremia is related to the accumulation of endogenous waste products considered to be toxins [2], followed many years later; second, by the recognition that dialysis treatment resulted in a remnant syndrome [3]; and third, more recently, by the recognition that what we have identified as uremic toxins may be components of an extensive signaling system [4] (Figure 1).

## 2. The Nascence of the Uremic Intoxication Paradigm

For many centuries, the prevailing paradigm in Western medicine has been that of Hippocrates and Claudius Galenus (also known as Galen of Pergamon). They theorized that the body consists of four humors (i.e., black bile, yellow bile, blood, and phlegm). Galen is credited with the idea that the kidneys are significant to the human body. Interested in blood circulation, he noted the disproportionally large blood supply of the kidneys [5]. As he could only rely on visual inspection and did not have access to microscopy, he was unable to decipher the actual workings of the kidneys, and so the role of the kidneys remained speculative [5].

It was not until the Renaissance that our understanding of the form and function of the kidney advanced. Bartolomeo Eustachio (1514–1574) wrote a treatise on the kidney, ‘De renum structura, officio, utilitate, et administratitione’ (‘On the structure, action, use and regulation of the kidney’), published in 1564 [6]. Scholars interested in 20th century kidney physiology will recognize parallels with the works of Homer W. Smith and his efforts to integrate knowledge in renal physiology, resulting in his magnum opus ‘The kidney—structure and function in health and disease’, published in 1957 [7].

Contemporaries of Eustachio included Fernel, one of the founding fathers of physiology, and Van Helmont. Jean Fernel declared that the kidneys, located beside the inferior caval vein, were suited to the extraction of serum from the blood (ouron, urine) [6]. Jan Baptist Van Helmont focused on lithiasis and explored the composition of urine. Much credit, however, goes to the Dutch physician Herman Boerhave, who was the first to develop and report a method to purify the ‘sal nativus urinae’ (the native salt of the urine) [8]. This salt later became known as urea, an end-product of protein metabolism in man.

It took until the nineteenth century for a London-based physician, Richard Bright, to describe a cohort of individuals with ‘dropsy’ (swelling due to edema), an increased bleeding tendency, visual disturbances, convulsions, and coma, ultimately leading to the death of those affected, a clinical syndrome that we recognize today as uremia [9]. In 1839, Robert Christison studied individuals with the advanced stage of “Bright’s disease”. He classified certain symptoms as “primary”, meaning that they were directly associated with the particular kidney condition. Others including digestive and neurological problems were considered to be “secondary” symptoms associated with renal failure and endogenous intoxication [10]. Theodor Friedrich von Frerichs, a German physician, studied a number of patients with “Bright’s disease”. In 1851, he coined the term ‘uremic intoxication’, combining the symptoms with his understanding of organic chemistry as he studied changes in blood composition and found an increase in urea and uric acid [2,11]. As urea could be readily measured at that time, several researcher-physicians sought to remove the substances believed to cause uremia by allowing a patient’s uremic plasma to equilibrate across a semipermeable membrane with a solution of sodium, chloride, and potassium, approximating the concentrations in normal serum [12].

Georg Haas is generally acknowledged as the scientist who—in 1924—was the first to conduct such an extracorporeal dialysis on a patient [13]. This experiment lasted only 15 min and was without complications. A second dialysis attempt followed in 1925 and lasted only 30 min. Four further experiments followed in 1926 [14]. Willem Kolff, a Dutch physician working in a small hospital outside of Amsterdam during World War II, saw many patients die with uremic symptoms. After brief attempts to develop an animal model of uremia, he undertook the treatment of patients with kidney disease and clinical evidence of uremic syndrome. The patient’s blood was exposed to a solution consisting of electrolytes and glucose across a semipermeable membrane that he estimated would allow for the passage and removal of small molecules considered to be possible uremic toxins [15]. He reported evidence of clinical improvement, in some instances quite dramatic, but always transient, until he reported sustained improvement in his fourteenth patient, who, it was later discovered, had sustained a reversible renal injury [16,17]. Kolff is considered the father of hemodialysis as we know it today. Kolff and others were not able to identify which of the solutes removed during hemodialysis accounted for the clinical improvement, but the clinical response to hemodialysis was seen as an effective treatment for renal failure, leading to the general belief that most uremic solutes were small enough to pass across synthetic membranes over the range of porosities employed in dialysis cartridges [18,19]. Urea and creatinine were seen as the measurable representatives of this group of uremic toxins. However, at the time, the exact nature of these toxins was not known, and it took until the introduction of advanced analytical methods such as chromatography and later mass-spectrometry for the detection of other toxins [20].

The National Cooperative Dialysis Study (NCDS) and hemodialysis (HEMO) study established Kt/V (K—dialyzer clearance of urea, t—dialysis time and V—volume of distribution of urea, approximately equal to patient’s total body water) as a marker of dialytic urea removal [21,22,23]. At the same time, the available data suggested that Kt/V_urea_ was more a marker of ‘inadequacy’ than of ‘adequacy’, and might not tell the whole story [24,25]. The studies performed in this first era of uremia research demonstrated the threshold effects of the dialytic clearance of small, water-soluble molecules. As shown by the early experimental treatments delivered by Haas and Kolff, there appeared to be no appreciable benefit from removing minor amounts of urea (and other small molecules). Kolff started to increase the amount of dialysis, thereby removing more and more solutes and, above a certain threshold, this kept the patients alive. With the advent of more efficient devices came the question of whether there was a saturation effect of this clinical benefit. The NCDS suggested a threshold effect with respect to the dialytic clearance multiplied by time, which was confirmed by the HEMO study. The interpretation of these studies was (and is) that the clinical effects of the dialytic clearance of small, water-soluble solutes plateaus, and that further increments in the dialytic clearance of small molecules do not lead to additional improvements in patient survival. 

Of note, one of the first dialysis sessions reported by Kolff as a successful and live-saving therapy decreased the blood urea concentration from 396 mg/dL to 121 mg/dL. This amounts to a urea reduction ratio of 0.69, and an estimated single pool Kt/V_urea_ of 1.17. Although this falls short of the current administrative threshold for reimbursement in the United States, it remains a magnificent achievement during the difficult times in which Kolff was working. 

## 3. The Remnant Toxins Era

While the focus was mostly on urea, it was well-known that dialysis removed significant amounts of other solutes. It was also recognized that while uremic symptoms abated, patient survival was limited, and complications still occurred. Babb and Scribner developed the middle molecule hypothesis in 1965, suggesting that the retention of solutes with a molecular weight exceeding 500 Da caused polyneuropathy. These molecules, thought to be larger than urea, were difficult to remove by hemodialysis. Hemodialysis was limited at that time to relatively short sessions with low surface area and small pore dialysis [26].

Epidemiologic analyses in the U.S. and abroad revealed an average life expectancy of roughly two and a half years, with uremic patients dying not of metabolic acidosis or other uncontrolled metabolic disorders, but rather of cardiovascular disease [27,28,29]. This finding provided the impetus to seek other “uremic toxins” whose removal by hemodialysis might not be estimated by the measurement of urea or creatinine. Review of the medical literature and subsequent laboratory confirmation of many suspect toxins by Vanholder and the European Uremic Toxin Work Group [3,30,31,32] identified many potential toxins that were classified as low molecular weight (e.g., urea and creatinine, <0.5 kDa), readily removed by conventional hemodialysis; middle molecules (β2-microglobulin and α1-macroglobulin, 0.5–58 kDa); and high molecular weight molecules too large to cross a glomerular basement membrane (large peptides like albumin, >58kDa). Vanholder and his colleagues focused their attention on a fourth category of toxins, namely low molecular weight solutes bound to a carrier protein, most often albumin, which they reasoned might be actively passed across cell membranes by “transporters” in normal renal tubular epithelium, but were not readily transported across the membranes employed in hemodialysis cartridges [33,34,35,36]. For an overview of the different classes of uremic toxins, we refer to a recent review by Rosner et al. [37]

These insights were consistent with earlier observations by Jared Grantham, who documented fluid and electrolyte transport (secretion) in isolated murine proximal tubules [38]. He observed a differential effect of serum from healthy donors versus patients with uremia [39] added to the perfusate [40,41]. It also aligned with data from Japan, where Toshimitsu Niwa focused attention on the protein-bound solute indoxyl sulfate [42] and cresols [43] in a murine model of renal injury. In 1994, Niwa and his colleagues reported that indole, a product of gut metabolism, administered to rats with subtotal nephrectomy, developed progressive glomerular sclerosis [44]. They recognized that indole was converted into indoxyl sulfate (IS), which they identified as a uremic toxin. 

IS is highly protein-bound, predominantly to albumin [45]. In the majority of diseases, when renal function declines, the concentration of these protein-bound solutes increases. Early in the course of progressive renal disease when the concentration of these solutes is only modestly increased, unbound solutes may be removed by hemodialysis. Over time, with progression of the underlying renal disease, removal by hemodialysis cannot prevent the further accumulation of protein-bound solutes, and at this point, the effects of further accumulation become manifest at extrarenal sites. 

The clinical evolution, predominantly the frequency of cardiac failure, in patients undergoing protracted years of hemodialysis [46] suggests a possible effect of uremic toxin acting on vascular endothelium in extrarenal tissues. Several epidemiological studies have indicated the role of remnant uremic toxins, in particular the protein bound IS and PCS [47]. IS is associated with cardiovascular mortality [48], cardiovascular events [49], congestive heart failure [50], and vascular access thrombosis [51].

Studies of umbilical vein endothelial cells by Gondouin et al. [52] described the effects of IS on the expression of 50 genes including genes involved in endothelial function and atherosclerosis. Several lines of evidence suggest the transport of protein-bound toxins such as IS and PCS across the membranes of vascular smooth muscle as the underlying defect responsible for increased vascular smooth muscle tone in patients with uremia or advanced renal disease [53]. IS induced oxidative stress in vascular tissues with the inhibition or reduction in NO production, which is an important regulator of vascular tone [54,55,56]. Furthermore, uremic toxins contribute to the loss of cell–cell junctions, increasing permeability with the activation of signaling pathways including the aryl hydrocarbon receptor (AhR), nuclear factor kappa B (NF-κB), and mitogen-activated protein kinase (MAPK) pathways [57,58].

Endothelium is not the only cell type in which there is a proven effect of protein-bound uremic toxins. Vaziri demonstrated that the expressions of claudin-1, occludin, and ZO-1 in the colonic mucosa of CKD rodents [59,60] and tight-junction forming human enterocyte (T84-cells) cultures in medium enriched with the serum of hemodialysis patients and theorized that this was the result of protein-bound uremic toxins [61]. In hepatocytes, there is reported oxidative stress of IS and a change in mitochondrial function [62]. In human hepatoma cells (HepG2), IS increases the expression and activity of the efflux transporter P-glycoprotein (P-gp) encoded by ABCB1 without modifying the expression of the other transporters. This effect is dependent on the AhR pathway [63].

At the level of the blood–brain barrier, we see similar effects of protein-bound uremic toxins, and more specifically, IS [64]. Epidemiologically cognitive function impairment and plasma IS concentration is correlated [65]. In vitro studies have demonstrated IS accumulation in brain tissue [66,67] and that IS has the ability to interfere with the blood–brain barrier in rat models with CKD, with this effect being dependent on the AhR pathway [68].

Several studies showed an increase in systemic inflammation, and more specifically, a connection with indoxyl sulfate in kidney disease [69,70,71,72]. Vaziri investigated the expression of pro-inflammatory transcription factors in kidney tissue such as nuclear factor kappa-light-chain-enhancer of activated B cells (NF-B) and NF-E2–related factor 2 (Nrf2) [73]. Of particular interest here is the role of macrophages [74,75]. Sun showed that the intestinal macrophages of uremic rats were activated in intestinal tissue. They had fewer cytoplasmic protrusions and pseudopodia, decreased electron density, and a greater number of organelles (especially lysosomes) from ruptured macrophages in the intercellular space; they speculated that this could contribute to the translocation of microorganisms [76]. IS increased inflammation and oxidative stress in IEC-6 cells and showed pro-inflammatory and pro-apoptotic properties in the peritoneal macrophages of mice with chronic renal disease [77]. The study of in vitro macrophage cell culture (RAW 264.7) showed a stimulation of NF-κB mRNA expression by IS, while Nrf2 was downregulated [72]. Lai and colleagues demonstrated once again the significance of the Nrf2/NF-κB signaling pathway in macrophages generated from monocytes [78]. Hemodialysis serum activated the AhR and enhanced TNF- production in the monocytes, resulting in a proinflammatory shift fromclassic to nonclassic and intermediate monocytes [79]. OATP2B1 (SLCO2b1) appears to be a potential transporter, as suppression using siRNA decreased these effects in peritoneal macrophages [80].

The overarching concept based on epidemiological and experimental data thus became that dialysis removed most but not all of the uremic retention solutes. It was postulated that uremia was the sum of residual toxic substances. During this second phase of uremia research, the aim was to identify and enumerate these residual toxins. A better understanding of the physicochemical properties revealed some molecules that are not easily removed using conventional dialysis techniques. 

## 4. Remote Sensing and Signaling

Over time, there has been an increasing emphasis on the production and transport of these challenging-to-remove solutes. The role of colon microbes in producing IS was first described by Brummer and Kasanen in 1955 [81]. Later, HPLC verified the colonic origin of p-cresyl sulfate (PCS) and IS by comparing colectomy patients with CKD to patients whose intestines were structurally intact [82]. It is now recognized that tryptophan and phenylalanine, protein breakdown in the colon, are filtered across the intestinal epithelium, converted to indoxyl and tyrosine, and translocated to the liver where they undergo sulfation and hydroxylation, which are taken up again by the kidney as IS and PCS [32].

The fact that small molecules bound to a larger protein are readily excreted by normal renal tubules led to the recognition that transport must be facilitated by transporters along the luminal or basolateral membranes of the renal tubule [83]. Organic anion transporters (OATs) like OAT 1 (SLC22A6) and OAT 3 (SLC22A8) were identified on the basolateral membrane and OAT 4 (SLC22A11) on the apical membrane of renal tubules [84,85], which have been shown to play a role in the transport of these toxins. Similarly, cationic transporters have been reported to mediate the release of inflammatory cytokines in conditionally immortalized renal proximal tubular epithelial cells [86,87]. OATs and organic anion transporter polypeptides (OATPs) are members of the SLC family. OATs belong to the SLC22A superfamily, which consists of six subfamilies, namely OAT, OAT-like, OAT-related, organic cation transporter (OCT), organic cation/carnitine transporter (OCTN), and OCT/OCTN-related. They mediate the transport of mainly organic anions across the cell membrane and play an important role in the absorption, distribution, metabolism, and excretion (ADME) of drugs [83,88,89]. Many [30] studies have recognized that several of these poorly dialyzable protein-bound solutes, notably IS and PCS, accumulate as nephron function declines and likely represent uremic toxins. Just as uremic toxins require specific transporters to pass from the lumen of the nephron to the peritubular space, it has become evident that specific transporters are a component of the extrarenal actions of some of these protein-bound solutes [90]. 

Although there is a significant amount of information available regarding the transport mechanisms in the proximal tubule, there is limited evidence regarding the particular transport of uremic toxins in other types of cells. Concerning intestinal transport, Morimoto and colleagues examined the ex vivo IS production in rats with adenine-induced CKD and demonstrated that it was excreted in the intestinal lumen. Despite interacting with MRP2 and BCRP, IS was not a substrate of these intestinal ABC efflux transporters. p-Aminohippuric acid effectively reduced the absorption-dominated IS transport in Caco-2 cell monolayers, indicating a comparable efflux pathway to that of the proximal kidney in rodents [91]. However, in humans, these mechanisms must be different, since the expression of these transporters is non-existent in the gut epithelium [92,93]. Any information on the transport of precursors like indole is virtually non-existent. Hepatocytes play an important role in the pathway of IS once indole is absorbed across the intestinal epithelial cells into the blood. After the uptake of this compound, they are metabolized by the hepatic microsomal cytochrome P450 family 2 subfamily E member 1 (CYP2E1) and the sulfotransferase family 1A member 1 (SULT1A1) to form IS. CYP2E1 is a phase I detoxification enzyme within the liver and is responsible for metabolizing ethanol and catalyzing the 3-hydroxylation of indole in hepatic microsomes, subsequently, 3-hydroxyindole (indoxyl) is sulfated by SULT1A1 to form 3-indoxylsulfate and 3-indoxylsulfuric acid [88,89]. Since the liver expresses transporter like OAT2 and OAT4, we can infer a similar transport mechanism as in the proximal tubule, however, the details are not known. 

It has been suggested that transport across vessels of uremic toxins like IS also regulate signaling and metabolism, potentially affecting gene expression in extra-renal tissues as well as the kidney. It seems reasonable at this time to speculate that the findings of fibrosis and functional disorders in extrarenal tissues and organs in advanced renal disease and uremia suggest a far more general role for small molecules such as IS. Focusing on the history and evolving story of IS and several other protein-bound molecules, currently viewed as potentially toxic metabolites eliminated via organic anion or cationic transporters, some of these small molecules appear to function as messengers acting on renal or extrarenal targets. Sanjay Nigam has proposed that uremic toxins are part of an extensive “remote sensing and signaling” network involving transporters and enzymes that modulate metabolism and signaling [65]. Viewed in this way, it seems likely that there might be some aspect of the remote signaling system that accounts for modulating the system in response to altered stimuli (e.g., to changes in fluid balance, diet, or physical activity). We suggest that this modulation might be analogous to the role played by check point inhibitors in the regulation of cell growth and proliferation. Check point inhibitors permit changes in critical molecules to influence (increase or decrease) the expression of a large array of target molecules. We suggest the possibility that “uremic toxins” exert a comparable control of the expression of a “remote signaling system” controlling fluid and electrolyte metabolism. 

## 5. Treatment of “Uremia”

With conventional dialysis treatments, uremia persists as a remnant syndrome. Considering that these compounds attached to proteins persist even after hemodialysis, it is possible that better procedures may be available to effectively remove them. As previously stated, advancements in dialysis techniques have improved the elimination of small water-soluble molecules. When it comes to middle molecular weight molecules, there have been advances using specialized dialyzers that have larger pores on the membrane surface (high-flux membranes) and convective hemodiafiltration (HDF). This has been the cause of some controversy. A comprehensive review conducted by COCHRANE in 2015 comparing convective dialysis methods found inadequate evidence to reliable conclude on the treatment’s effects on major clinical outcomes. It was determined that these methods may reduce cardiovascular mortality but not all-cause mortality [94]. Randomized controlled trials (RCTs) have assessed the effect of HDF on mortality rates in different countries. Notable trials include the Dutch Convective Transport Study (CONTRAST) [95], the Comparison of Post-dilution Online Hemodiafiltration and Hemodialysis (Turkish OL-HDF) Study [96], the Estudio de Supervivencia de Hemodiafiltración Online (ESHOL) study [97], and the French Convective versus Hemodialysis in the Elderly (FRENCHIE) study [98]. While the results of these studies were underwhelming, the recent CONVINCE study demonstrated convincing evidence of reduced all-cause mortality when comparing high-efficiency HDF versus traditional hemodialysis [99]. Further discussion of these findings was out of the scope of this article; however, we assert that these methods will consistently encounter challenges in eliminating protein-bound compounds such as IS and PCS. As far as we know, none of these randomized controlled trials (RCTs) assessed the impact on IS or PCS. Small observational trials found no significant difference in the clearance of IS [100,101,102]. Even if we were to assume the existence of a flawless dialysis technique capable of efficiently removing small- to medium-sized molecules, it would still be ineffective in eliminating protein-bound uremic toxins such as IS and PCS. These molecules primarily bind to albumin; when necessary, large proteins like albumin are eliminated, which can lead to a deficient state characterized by malnutrition and hypoalbuminemia [103].

Another option is to increase the rate of diffusion over the membrane. This can be achieved by ensuring a consistent difference in concentration across the dialysis membrane. There are two options available in this case: adding a sorbent to the dialysate to effectively remove indoxyl sulfate, or increasing the flow of dialysate to maintain a low concentration. Proof-of-concept studies with either activated charcoal in dialysis or very high dialysate flows were able to effectively increase the removal of protein-bound uremic toxins both in laboratory experiments and in living organisms, where there have been multiple reviews on this subject, even in this journal [45,104]. However, the effect on clinical outcomes in this setting have been limited to small trials.

If the clearance of these molecules is not an option, we could look into their production in the colon by the microbiome. There are currently few therapeutics targeting the absorption of gut-derived uremic toxins directly. Since these protein-bound toxins are hard to dialyze, it could be an option to target the production of these compounds. We are aware of one commercially available therapeutic, namely AST-120 (or Kremezin, consisting of oral, spherical carbon particles that adsorb uremic toxins and their precursors in the gastrointestinal tract, currently marketed in Japan, Korea, Taiwan, and the Philippines) [105]. While some evidence exists on the effect of the concentration of uremic toxins [106,107,108], these were not always reproducible, and the effect on clinical endpoints remains sparse [109].

Another option is to target the producing microbiota of the gut-derived uremic toxins. While there is evidence in preclinical models, an effect in humans is still lacking. Certainly, some intermediate outcomes have been reported. Guida demonstrated the potential of inulin (a prebiotic) combined with probiotic therapy on plasma p-cresol as early as 2014 [110]. Multiple studies have demonstrated the potential to affect the gut flora of CKD patients with pre- [111,112] and probiotics [113,114]. These changes were often connected to a decrease in serum uremic toxin level [115,116,117]. Not all studies could prove an effect on the serum uremic toxins, however [111,118,119,120], or showed statistically significant but clinically minimal changes [121]. There are, however, almost no studies looking at disease specific outcomes. De Mauri et al. found a trend toward dialysis-free survival in non-dialysis CKD stages 3 and 4 patients; however, this trend was not statistically significant [122]. Concerning the patient-specific endpoints, there is conflicting evidence; the original Guida research did not demonstrate that symbiotics alleviated gastrointestinal problems in CKD patients [110]. Belova showed an increase in the self-reported quality of life after symbiotic treatment [123] while Cosola and colleagues looked at the amelioration of abdominal pain and constipation syndromes in CKD patients after synbiotics [124]. We refer to two recent meta-analyses that looked at the effect of dietary interventions (pre- and probiotics) on inflammatory factors, uremic toxins, and gastrointestinal symptoms in patients undergoing dialysis [125,126].

Given the current data, we are unable to provide any particular therapy advice. However, we can indicate other areas of research that warrant further investigation. First, it would be intriguing to evaluate the efficacy of removing uremic toxins in the large HDF RCTs and determine whether certain subgroups can be identified where the enhanced clearance of IS/PCS leads to improved survival. Additionally, further therapeutic alternatives for purification should be thoroughly examined in large-scale clinical trials. Nevertheless, we acknowledge that budgetary constraints may be an issue. Furthermore, it is necessary to evaluate the impact of inhibiting the absorption of gut-derived uremic toxins that are bound to proteins in the colon on clinical outcomes, and not just intermediate outcomes. Finally, in accordance with the remote sensing and signaling concept, we propose that the primary objective of treating uremia should be to optimize the imbalanced signaling cascades by reducing the clearance of these signaling molecules. However, further in vitro study is necessary to investigate the precise mechanisms by which these compounds affect somatic cells. 

## 6. Conclusions

**Figure 2:**
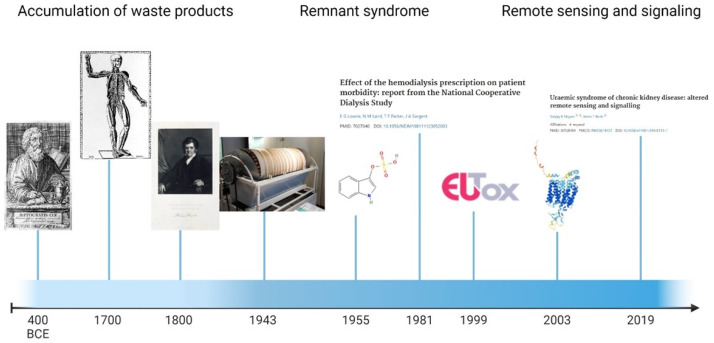
From left to right: Portrait of Hippocrates from Linden, Magni Hippocratis 1665 Wellcome Library, London; Vascular system from Bartolomeo, Eustachio T‘abulae Anatomicae’, edited by J.M. Lancisi, 1714 Wellcome Library, London; Portrait of Richard Bright, 1838, Great Norwegian Encyclopedia Bergen; Photo of Kolff’s artificial kidney, Museum Boerhaave, Leiden; Chemical structure of indoxyl sulfate, PUBCHEM, National Library of Medicine; Publication of NCDS study [23], PUBMED, National Library of Medicine; European uremic toxin group logo; 3-dimensional structure of OAT3 (SLC22A8), GeneCards, The Human Gene Database; Publication of uremic syndrome of chronic kidney disease [4], PUBMED, National Library of Medicine. All images available under Creative Commons license.

The concept of uremia has undergone changes and development throughout history (Figure 2). Starting with the first notion of the four humors by Hippocrates and Galen, our comprehension of kidney structure and function was developed during the Renaissance by Bartolomeo Eustachio, Fernel, Van Helmont, and Boerhave. The concept of uremia as intoxication caused by internal substances was introduced by Bright and Christison. Dialysis provides a method for eliminating these toxins from the body. In the first half of the 20^th^ century, the German physician Georg Haas successfully conducted a 15-minute extracorporeal dialysis procedure, and the first clinically successful dialysis session was conducted by the Dutch physician Willem Kolff. However, even with advances in modern dialysis, there is a remnant clinical syndrome. Dialytic clearance of water-soluble solutes is not enough, and further increasing the dialytic clearance of these small molecules does not enhance patient survival. 

The existence of protein-bound molecules is the reason for the lack of effectiveness as these are not readily removed using our current methods of dialysis, and the removal of these substances cannot be measured using urea or creatinine. Protein-bound IS and p-cresol are common toxic substances seen in the blood of individuals with kidney failure that are primarily attached to albumin. They are produced in the intestines through the breakdown of amino acids such as tryptophan and phenylalanine by bacterial fermentation. These uremic toxins build up as kidney function declines and the levels of waste substances increase, and are responsible for the various complications of kidney disease. 

Remote sensing and signaling hypothesis might explain the central role these compounds play in disease, altering gene expression in the kidney and extra-renal tissues. These tiny molecules may communicate with renal or extrarenal targets as part of a “remote sensing and signaling” network. However, there are still multiple processes that need to be clarified. The transport of protein-bound uremic toxins in the intestinal epithelium, vascular endothelium, and hepatocytes is still not well-understood as well as the method by which these toxins affect specific tissues. While treatment techniques have shown effectiveness in in vitro models, preclinical animals, and intermediate endpoints, their influence on clinical endpoints is still unknown.

## Data Availability

No new data were created or analyzed in this study. Data sharing is not applicable to this article.
